# Impact of Proton Therapy Implementation on Processes, Patient Satisfaction, and Technology Use in a Radiation Therapy Department

**DOI:** 10.1016/j.adro.2025.101988

**Published:** 2025-12-25

**Authors:** Luca Heising, Thijs Ackermans, Liesbeth Boersma, Carol Ou, Geert Bosmans, Rachelle Swart, Andre Dekker, Maria Jacobs

**Affiliations:** a Department of Information Systems and Operations Management, Tilburg School of Economics and Management, Tilburg University, Tilburg, North Brabant, The Netherlands; b Department of Radiation Oncology (Maastro), GROW School for Oncology and Reproduction, Maastricht University Medical Centre+, Maastricht, Limburg, The Netherlands; c Department Strategy and Entrepreneurship, Tilburg School of Economics and Management, Tilburg University, Tilburg, North Brabant, The Netherlands

## Abstract

**Purpose:**

Because proton therapy (PT) can be regarded as a major radical innovation, its implementation in a radiation therapy (RT) department may have an adverse impact on processes. The current study aimed to investigate the effect of PT implementation on disruptions of clinical processes, patient satisfaction, and technology use.

**Materials and methods:**

The study was performed in an independent Dutch RT institute, where PT was implemented in February 2019. Endpoints were (1) process disruptions, (2) patient satisfaction, and (3) technology use. Causal inference and Wilcoxon rank-sum tests in R and MATLAB were used for the analyses.

**Results:**

After the implementation of PT, human-related errors and organizational culture-related errors in the photon therapy (PhT) process increased. Our empirical data showed more process disruptions associated with PT than with PhT. The implementation did not significantly affect patients’ satisfaction. Analysis of technology use showed a decrease in PT uptime, including treatment stagnations lasting ≥2 days. The organizational process of the entire clinic was affected in the first 13 months after PT implementation because of significantly more process disruptions in PT compared to PhT and an increase in some distinct PhT process disruptions. The decrease in PT machine uptime below 95% caused treatment stagnation with consequences for patients and staffing.

**Conclusions:**

This study provides the first quantitative assessment of introducing PT in an ambidextrous, ambitious center that also runs PhT. Incident profiles shifted toward human-related errors in PhT and organizational issues in PT, with no lasting change in patient satisfaction. In conclusion, successful adoption requires stronger preparation and training, early patient engagement, and proactive planning for quality control, frequent updates, and lower initial uptime in partnership with vendors.

## Introduction

Since 2018, proton therapy (PT) has been accessible to patients with cancer in the Netherlands, with 3 out of 19 radiation therapy (RT) departments offering this treatment. At the end of 2022, a total of approximately 3550 patients received PT, and 75% of the available yearly capacity of 1600 patients was achieved.^1^ One of these PT centers successfully integrated a complete PT infrastructure into an existing RT center.^2^ The advantages of this concept are that several devices, such as imaging and supporting services, can be shared with the existing RT center, resulting in lower costs. Also, there are logistical and operational advantages for employees working in both photon therapy (PhT) and PT. However, there are also potential disadvantages because PT can be considered a radical innovation (RI) (Appendix E1). In such an integrated concept, ambidextrous skills are required during the implementation process of RIs as PT^3^^,^^4^: that is, different competences are required to operate both the regular PhT efficiently and effectively and to simultaneously implement PT (eg, dealing with uncertainty because of changing protocols “under construction,” new techniques, adopting new knowledge and skills, new referral relations, etc). Previous work found that applying an ambidextrous approach in such situations, in general, can be beneficial for the improvement of health care quality with simultaneous cost containment,^5^ because both the regular operations (PhT) and the RI (PT) draw on similar scarce resources. Furthermore, additional innovation challenges arise because of the different characteristics of care delivery.^6^

Little is known yet about the actual impact of RI on regular health care operations in an ambidextrous setting, despite the wide recognition of this impact.^7^ Therefore, we aimed to scrutinize the impact of the PT implementation in an integrated PhT-PT facility by comparing the process disruptions, patient satisfaction, and technology use, that is, uptime, updates, and quality control checks, between PT and PhT.

## Methods and Materials

### Case setting

The study was carried out in an independent RT center in the Netherlands, where PT was implemented in 2019 and fully integrated into the existing infrastructure for PhT. The implemented PT technology consists of a compact single-room facility with an innovative PT machine with many new features that had to be explored. Equipment was delivered by different vendors, leading to higher complexity in maintenance and regulation. Although there are several centers in the US with a similar infrastructure, it was the first implementation of this infrastructure in Europe.^1^ The RT center treats approximately 4000 patients per year with PhT, with 6 linear accelerators. For PT, a license is granted to treat 400 patients a year with 1 PT machine. This study described the first 13 months of operation. The center had decided to scale up in 3 years with a scheme of respectively 117, 233, and 350 patients per year, respectively, and ultimately 29 full-time equivalents across all roles. The RT center employs approximately 330 staff members in total. In the clinic, most employees working on PT were recruited from the PhT site. Especially in the first phase, the employees underwent a thorough training program. After onboarding of the first staff, PT training required gradual onsite training on the PT machine by in-house staff and an e-learning module, followed by an examination. Complementary to the onsite training and e-learning, site visits to other PT centers were arranged. For the physics commissioning of the system, external proton experts were hired.

### Measurements

#### Endpoints

For both conventional PhT and PT, we hypothesized that the implementation of such a RI had a negative effect on (1) the stability of clinical processes, which we define as the predictability and consistency of processes measured through error rates, inspired by Ramirez and Runger^8^ (2006); (2) patient satisfaction, since the implementation of PT may have distracted employees from their routine PhT operations and service to patients; and (3) technology use, that is, uptime, additional updates and quality controls, and thereby daily routine. Therefore, we analyzed 3 endpoints: patient process disruptions, patient satisfaction, and finally, technology use.

Patient process disruptions were measured using the existing incident reporting system, based on the PRISMA-Medical Eindhoven classification model.^9^ The incidents are categorized into 4 main root cause categories: technical (T), organizational, human, and patient-related (Table 1). These categories are again divided into 19 subcategories. Each incident is documented under one of the subcategories with their corresponding indices (eg, root cause T in combination with category external (EX) results in T-EX) by trained safety and quality employees. Patient-related incidents are incidents that are directly related to the patient and cannot be influenced by the organization, such as concurrent medications prescribed by EX providers that interact with radiation (eg, radiosensitizers) without the treating center being informed. Patient-related incidents were omitted in the analysis because the organization has no direct influence on these types of incidents. Additionally, the clinical process does not remarkably differ in complexity between PT and PhT besides the steps required for plan comparisons (Appendix E2). The number of root causes of incidents was compared in 2 ways: (1) within PhT, before and after the implementation of PT, and (2) between PT and PhT, both before and following PT implementation.

**Table 1:** Table root errors categories, subcategories, and explanation based on Snijders et al^9^

Root error		Code	Category	Definition
T		T-EX	EX	Technical failures beyond the control and responsibility of the investigating organization.
		TD	Design	Failures because of poor design of equipment, software, labels, or forms.
		TM	Materials	Correct design, which was not constructed properly or was set up in inaccessible areas.
O		O-EX	EX	Failures at an organizational level beyond the control and responsibility of the organization, such as in another department or area.
		OK	Transfer of knowledge	Failures resulting from inadequate measures taken to ensure that situational or domain-specific knowledge or information is transferred to all new or inexperienced staff.
		OP	Protocols	Failures relating to the quality and availability of the protocols within the department (too complicated, inaccurate, unrealistic, absent, or poorly presented).
		OM	Management priorities	Internal management decisions in which safety is relegated to an inferior position when faced with conflicting demands or objectives. This is a conflict between production needs and safety. An example of this category is decisions that are made about staffing levels.
		OC	Culture	Failures resulting from a collective approach and its attendant modes of behavior to risks in the organization.
H		H-EX	EX	Human failures originating beyond the control and responsibility of the organization. This could apply to individuals in another department.
	Knowledge-based behavior	HKK	Knowledge-based behavior	The inability of an individual to apply their existing knowledge to a novel situation. Example: a radiation therapy technologist who is unable to deal with unknown artifacts during contouring.
	Rule-based behavior	HRQ	Qualifications	The incorrect fit between an individual’s training or education and a particular task. Example: expecting a technician to solve the same type of difficult problems as a technologist.
		HRC	Coordination	A lack of task coordination within a health care team in an organization. Example: an essential task is not being performed because everyone believes that someone else has completed the task.
		HRV	Verification	The correct and complete assessment of a situation, including related conditions of the patient and materials to be used before starting the intervention. Example: failure to correctly identify a patient by asking their birthdate.
		HRI	Intervention	Failures that result from faulty task planning and execution. Example: acquiring CBCT scan, although not scheduled.
		HRM	Monitoring	Monitoring a process or patient status. Example: proceeding with treatment while pretreatment was not accepted.
	Skill-based behavior	HSS	Slips	Failures in the performance of highly developed skills. Example: computer entry error of a well-performed task.
		HST	Tripping	Failures in the whole body movements. These errors are often referred to as slipping, tripping, or falling.
Other factors		PRF	Patient-related factor	Failures related to patient characteristics or conditions, which are beyond the control of staff and influence treatment.
		X	Unclassifiable	Failures that cannot be classified in any other category.

*Abbreviations:* CBCT = cone beam computed tomography; EX = external; H = human; H-EX = human extrnal; HKK = human knowledge based behavior; HRC = human rule-based behavior coordinatin; HRI = human rule-based behavior intervention; HRM = human rule-based behavior monitoring; HRQ = human rule-based behavior qualifications; HRV = human rule-based behavior verification; HSS = human skill-based behavior slips; HST = human skill-based behavior tripping; O = organizational; OC = organisational culture; O-EX = organisational external; OK = organisational transfer of knowledge; OM = organisational management priorities; OP = organisational protocols; PRF = patient-related factor; T = technical; TD = technical design; TM = technical materials; X = unclassifiable.

Patient satisfaction was measured by analyzing feedback cards of patients treated with PT or PhT. All patients in the RT center are routinely offered the opportunity to fill out a feedback card, which is green (very satisfied), yellow (room for improvement), or red (something went wrong). Patients are immediately approached for a follow-up. In total, 474 feedback cards were received in the postimplementation phase. Because of missing data, it was not possible to trace back all the feedback cards to PT or PhT. We therefore omitted feedback cards without a label, leaving us with a sample of 254 cards for the analysis of the postimplementation phase.

Technology use was measured as an unexpected decrease in uptime and the number of additional quality controls. Uptime data for the equipment was collected monthly for both PT and PhT. Uptime was defined as the percentage of time the machine was in clinical use during its scheduled operational hours, excluding periods of planned inactivity. These data are systematically recorded by the RT center and was retrieved for the purposes of this study. Because the inactivity of the PT machine can lead to cancellation of the PT fraction and/or replacement of the PT fraction by a PhT fraction, we also measured treatment stagnations, defined as downtime with a duration of 2 days or longer. Lastly, we observed the frequency of updates or quality control checks for both PT and PhT to illustrate the effort and dedication needed for the technology. Because of the limitations inherent in retrospective data collection, we were unable to capture unplanned quality control checks triggered by downtime or hardware/software updates. These events could have provided additional insight into the extent of unplanned full-time equivalent effort required. However, such instances are largely reflected in the recorded downtime data.

#### Measurement periods

For PhT and for the endpoints process disruptions and patient satisfaction, we collected weekly data across a preimplementation period (January 2017-February 2019) and a postimplementation period (February 2019-December 2019) (Table 2). This provided a baseline measure for PhT, allowing us to assess whether the implementation of PT had any effects on PhT over time. For PT, data were collected monthly because the number of PT patients varied greatly in the early phase; some weeks had no patients at all, making weekly data less meaningful. PT data collection began 3 months after PT implementation (between May 2019 and May 2020, inclusive) to exclude the initial start-up phase, which was considered unrepresentative of routine clinical practice.

**Table 2:** Summary of data collection and data analysis

Data type	Unit of measure	Time period	Frequency	Description	Analysis
Process interruptions					
Incident reports	Count	May 2019 to May 2020	Monthly	Root errors for PT and PhT according to PRISMA documentation	Wilcoxon rank-sum test, comparing PhT with PT
Incident reports	Count	January 2017 to December 2019	Weekly	Root errors for PhT	Causal impact analysis, comparing PhT incidents before PT implementation and thereafter
Patient satisfaction					
Report cards	Count	January 2017 to December 2019	Weekly/monthly	Patients’ feedback cards (red/yellow/green)	Mann-Whitney U test
Technology use					
Downtime	Percentage	May 2019 to May 2020	Monthly	The duration for which the machine was expected to be used, but cannot be used	Descriptive
Software releases	Count	May 2019 to May 2020	Monthly	Amount of software releases per technology	Descriptive
Hardware updates	Count	May 2019 to May 2020	Monthly	Number of times hardware needed improvement	Descriptive
Quality control checks	Count	May 2019 to May 2020	Monthly	Number of times the quality of the machine was checked	Descriptive

*Abbreviations:* PhT = photon therapy; PT = proton therapy.

#### Data analysis

Because the number of fractions highly impacts all endpoints, we corrected for the number of fractions in the corresponding timeframe before performing the statistical analyses. The data to measure patient process disruptions were not normally distributed; we performed a Wilcoxon rank-sum test to compare incidents of PT data with PhT data. A causal impact analysis on PhT data only was performed by comparing the number of incidents and their causes before implementation of PT (ie, from January 2017-February 2019) with the period after implementation of PT (February 2019-December 2019). For this analysis, the expected number of incidents related to PhT after implementation was estimated based on the incident report data between January 2017 and January 2019 and corrected for the number of patients and subsequently compared with the actual number of incidents after the PT implementation date to get an indication of the effect of the PT implementation on PhT. Because of the number of statistical analyses, we corrected the *P* value with the conservative Bonferroni correction.^10^

The patient feedback cards were compared with a Mann-Whitney U test because the data were not normally distributed. The U statistic summarizes how often PT’s cards exceed PhT’s cards; ties count as 0.5 each. We compared patient satisfaction of patients treated with PhT with patients treated with PT from February 2019 to December 2020; in addition, we investigated whether patient satisfaction of patients receiving PhT treated before PT implementation, that is, January 2017-January 2019, differed from satisfaction after PT implementation.

Descriptive statistics were performed to get insight into technology use, that is, uptime, updates, and quality control for PT during the first 13 months of implementation (May 2019 up to and including May 2020).

All analyses were performed using R (R Foundation for Statistical Computing) and MATLAB (MathWorks). More specifically, for causal inference analysis, we used the causal impact library in R.^11^ We used the number of patients as a predictor variable, as this is not affected by the intervention. The Wilcoxon rank-sum tests were performed in MATLAB, version R2021a.

## Results

### Disruption of processes

**Figure 1:**
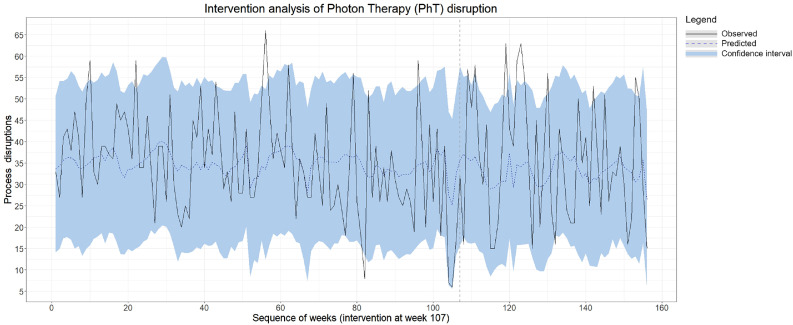
Causal Impact analysis of all photon therapy (PhT) process disruptions. The vertical gray dashed line indicates the implementation of proton therapy (PT). The black line represents the estimated counterfactual, and the blue dashed line is the observed number of patients in the predictor variable. The blue area represents the 95% confidence interval.

In the period before the implementation date of PT (January 2017-February 2019), we observed an average of 34.7 PhT process disruptions per week in the data. In the period after the implementation date of PT (February 2019-December 2019), we expected an average of 33.8 PhT process disruptions (95% confidence interval [CI], 30.1-37.4) if PT had not been implemented. The actual observed number of process disruptions in the period after the implementation date was 35.3 process disruptions per week. None of the disruptions were severe (ie, resulted in a 5% dose difference in the entire treatment). The causal impact analysis is illustrated in Fig. 1. Although this difference of 1.5 in the total number of process disruptions was not statistically significant (95% CI, −2.1 to 5.2, *P* = .2), the distribution of root causes shifted. We found significant increases in some subcategories of the root causes; that is, PT implementation had the largest impact on human errors: human rule-based behavior root causes increased by 23% with 0.75 incidents (95% CI, 2.7-3.9, *P* = .01), human knowledge-based behavior root causes increased by 44% with 0.77 incidents (95% CI, 21%- 68%, *P* < .01), and human EX was 108% higher than the expected number of root causes of incidents. Causal impact graphs for the main subcategories can be found in Appendix E3. Additionally, the organizational culture root causes of incidents increased significantly after the implementation of PT, with an effect of 0.7, from the expected 2.36 incidents on average (95% CI, 1.71- 2.97) to the observed 3.06 incidents (*P* < .01). Other organizational root causes of incidents and technical ones remained relatively stable.

**Figure 2:**
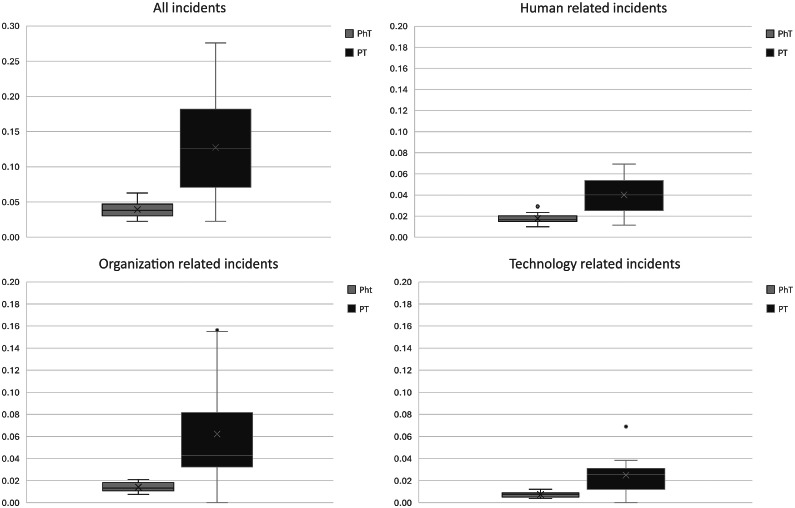
Photon therapy (PhT)-related incidents and proton therapy (PT)-related incidents relative to the number of fractions.

Significantly more process disruptions were seen for PT than for PhT for the 3 main categories: technology, human, and organization (Table 3). For all categories combined, the median difference of incident reports relative to the number of fractions receiving the treatment concerned was 0.09 higher for PT (*M* = 0.126) than for PhT (*M* = 0.038); this difference was significant with a moderate effect size (*Z* = 4.13, *r* = 0.344, *P* < .001). This difference can be seen in the upper left boxplot (Fig. 2). Of the main categories, organization-related incidents had the largest median difference (0.029).

**Table 3:** Wilcoxon rank-sum test comparison of root causes of process interruptions between proton therapy (PT) with photon therapy (PhT)

Root causes	*P*	Corrected *P* value	z	Rank-sum	Md PhT	Md PT	Md diff
TECH	.001	.016	−3.435	90	0.010	0.025	0.015
TD	.001	.016	−3.320	92	0.006	0.018	0.012
TC	.002	.032	3.052	199	0.000	0.000	0.000
TM	.015	.240	2.443	186	0.002	0.000	−0.002
Human	.000	.000	−4.128	78	0.017	0.040	0.024
HKK	.150	1.000	1.441	175	0.003	0.000	−0.003
HRC	.175	1.000	1.357	173	0.000	0.000	0.000
HRI	.000	.000	−3.782	84	0.008	0.031	0.022
HRM	.644	1.000	.463	158	0.000	0.000	0.000
HRV	.259	1.000	−1.128	130	0.004	0.008	0.004
Organization	.000	.000	4.128	222	0.013	0.043	0.029
OC	.582	1.000	−.551	140	0.003	0.009	0.006
OK	.174	1.000	−1.360	126	0.002	0.008	0.006
OM	.006	.096	−2.743	102	0.004	0.015	0.012
OP	.040	.640	−2.051	114	0.004	0.015	0.011
Total	.000	.000	4.128	222	0.038	0.126	0.088

*Note:* Columns: *P* value indicates significance, marked bold if below the .05 threshold, corrected *P* value was corrected with Bonferroni correction to account for the multiple analyses; z-value of the rank mean, a z-value closer to 0 indicates more evenly distributed ranks; rank-sum, indicates the sum of the rank scores; the median errors relative to the number of patients is notated with Md, where the last column shows the Md difference between the group.

*Abbreviations: HKK = human knowledge based behavior; HRC = human rule-based behavior coordinatin; HRI = human rule-based behavior intervention; HRM = human rule-based behavior monitoring; HRV = human rule-based behavior verification; OC = organisational culture; OK = organisational transfer of knowledge; OM = organisational management priorities; OP = organisational protocols; TECH = technical; TD = technical design; TM = technical materials.*

### Patient satisfaction

From January 2017 to January 2019, less than 1% of all patients filled out a feedback card. Fifty-six patients receiving PhT filled out a red feedback card (standard deviation (s) = 0.6), 769 patients filled out a green feedback card (s = 2.9), and 77 patients receiving PhT filled out a yellow card (s = 0.8). Although the mean differences were not statistically significant (red cards: *U* = 63.5, *P* = .617; green cards: *U* = 60, *P* = .483; yellow cards: *U* = 75, *P* = .861; Table 4), patients receiving PT seemed more eager to fill out a card, and patients receiving PT wrote relatively fewer green cards compared with the total number of cards than patients receiving PhT.

**Table 4:** Statistics of the feedback cards of patients from February 2019-February 2020

Feedback card	Photon therapy	Proton therapy
Red	M = 0.010; SD = 0.005	M = 0.088; SD = 0.120
Green	M = 0.033; SD = 0.011	M = 0.11; SD = 0.195
Yellow	M = 0.008; SD = 0.005	M = 0.091; s = 0.1123

Red card means: something went wrong, action required; yellow card means: room for improvement; green card means: patient is satisfied.

*Abbreviations:* M = median, SD = standard deviation.

We found an equal number of green cards in the preimplementation and postimplementation periods, apart from the week that PT was implemented: only in that week was a dip in green cards observed. In the trend analysis of the red cards, we saw an increase shortly before and after the implementation of PT. Because of the small sample, we cannot conclude that this increase affects the PT implementation.

### Technology use: Uptime, updates, and quality control

**Figure 3:**
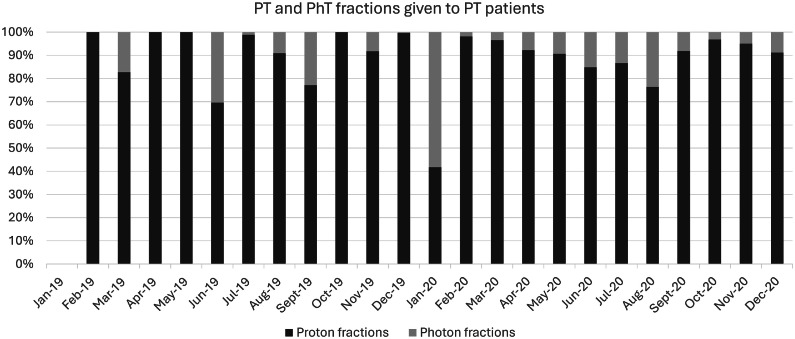
Percentage of fractions given with photons (light gray) and protons (dark gray) to patients meant to be treated with protons.

Despite the structural daily quality control checks and monthly hardware and software updates, a lower uptime than the expected 95% was observed. In 2019, the average uptime per month for a linear (photon) accelerator was 96.9%, with outliers down to approximately 85%; this applies to all 6 photon machines. The average uptime for the PT machine was 91.9% in 2019, with peaks of under 85%. From the implementation of PT to the end of 2020, the center experienced 5 treatment stagnations, with a duration of 2 to 7 days. During these periods, the RT center could not treat patients receiving PT, so their treatment was either postponed or replaced with PhT, depending on the RT schedule/indication and the urgency to complete RT within the maximal acceptable overall treatment time. Figure 3 illustrates the proportion of patients receiving PT treated with PhT. The larger the proportion of patients receiving PT treated with PhT within that month, the higher the impact of the stagnation on the entire RT clinic, because the disruptions also interfere with the PhT schedules.

To contextualize these uptime differences, the quality assurance (QA) framework and operational practice also differed between modalities. For protons, the American Association of Physicists in Medicine guidelines were followed because no local proton-specific guidelines were available at the time. For photons, the Netherlands Commission on Radiation Dosimetry guidelines were followed. Leveraging many years of experience with the photon equipment portions of the physics QA was streamlined under a strict protocol. Because every physicist was experienced with the photon systems and QA tools, routine photon QA could be executed efficiently by a single person.

By contrast, during early PT operations, a broader set of daily, weekly, and monthly constancy tests (eg, output, spot size, energies, symmetry, and flatness) was performed to build experience and confidence, although standard operating procedures were still being finalized and improved; all PT QA procedures were performed by at least 2 staff members. Over the 4-week analysis window, this ramp-up translated into substantially more time spent on QA for PT, approximately 7-fold compared with photons. In addition, the PT system required more frequent hardware and software updates. Although these were applied outside clinical hours, they occasionally reduced availability because several updates were first-in-world releases that required iterative troubleshooting.

## Discussion

This study quantitatively assessed the impact of PT as an RI implementation in health care in an ambidextrous context. Our findings showed that PT implementation in an integrated PhT/PT facility did not lead to more process interruptions in the already existing PhT, but the type of errors shifted toward more human-related errors. In addition, more unforeseen events arose in PT than in PhT, especially organizational incidents. Patient satisfaction showed a slight dip in the first week of PT implementation, but no persisting differences were seen in patient satisfaction postimplementation. The new technology caused a lower uptime than observed in the PhT but required more quality control checks and more frequent hardware and software updates.

### The impact on patient process disruptions

Most PhT incident categories remained stable after the implementation of PT, whereas none of the disruptions were severe (ie, resulted in a 5% dose difference in the entire treatment). The only incidents that were affected by the implementation of PT were human knowledge-based behavior, human rule-based behavior, and human EX behavior. Knowledge-based behavior incidents occur when the health care professional makes a mistake caused by a lack of knowledge of the procedure. A plausible explanation for the increment in knowledge-based errors in the PhT is that a small brain drain^12^ occurred in the PhT part of the department as skilled health care professionals were transferred to PT and new personnel were recruited for PhT. Rule-based behavior refers to incidents based on incorrect execution of the protocol. An increase in rule-based behavior can be caused by an increase in fatigue of employees because of stress or longer working hours. A recent review of the relationship between employee health and patient safety illustrated a negative outcome of employee fatigue and stress on their cognitive skills (eg, alertness and concentration) and changes in their behavior (eg, following safety procedures, focus, and communication).^13^ To ascertain fatigue and stress as a direct cause for the increase in rule-based errors found in this study, further research is required. Human EX errors are caused by people outside of the organization, for example, by the vendor; the organization typically has less impact on this.

When comparing PT to PhT, we found that more incidents relative to the number of fractions occurred associated with PT than with PhT. The root causes for the incidents were more often organizational-based than PhT-root causes, for which human-based errors dominated. This is possibly because when a process is stable, human root causes of process disruptions prevail.^14^ Organizational operations are more stabilized by policies, strategies, work procedures, and routines, which are underdeveloped when starting with an RI such as PT.

## The impact on patient satisfaction

The patient satisfaction remained relatively stable without any significant mean differences between patients treated with PT and PhT. There was a small dip in green cards in the week that PT was implemented, and there was an increase in red cards shortly before and after the implementation of PT. The fact that there were no major changes in patient satisfaction is in line with the absence of any severe disruptions and the fact that PhT incident categories remained stable after the implementation of PT. However, it should be noted that these findings should be interpreted with caution because the patients were not proactively asked to participate in an evaluation of their treatment and only represent the saturated cases in which the patient is extremely satisfied or extremely dissatisfied. For research building on the current study and as a recommendation for future research, patient satisfaction should be systematically measured through surveys and focus groups.

### Machine uptime after installation

The uptime of PT was lower than the critical threshold of 95% defined by the World Health Organization, which led to some treatment stagnation in the RT center. Among the factors exacerbating the amount of downtime were (1) the newness of the technology and software (first in Europe), (2) a lack of sufficient internal knowledge to remedy the downtime quickly, and (3) the RT center being the first to integrate hardware and software from different suppliers.^1^ A recent study with the aim of characterizing the initial experience of integrating an identical proton machine in the US described similar occurrences as experienced by the case described in the current study.^15^ It seems, however, that the performance in our study was a consequence of the completely new configuration. In 2022, the uptime performance was very good, above 97%, and comparable with older PT technologies.

### Lessons learned

One of the lessons learned was that although the overall disruption rates did not change significantly, one should be aware of potential differences in the root causes (ie, increased human rule-based errors, human knowledge-based errors, human EX errors, and organizational culture-related issues). Furthermore, it is important to incorporate patient feedback and engage them actively. Because treatment delays, which occurred in the present study, could induce increased psychological stress in patients, resulting in increased anxiety and depression, and have been seen at a different institution.^14^ In the present study, we experienced low response rates and low sample rates, which made it difficult to conclude the patient satisfaction outcome, which remained relatively stable. In terms of technology use, it is not realistic to expect similar behavior between 2 very different technologies (PT and PhT). Especially in terms of quality control checks and hardware and software updates, which will have a negative impact on the uptime. Finally, it is important to carefully consider in advance how personnel will be allocated or hired to support the new PT implementation. This ensures that the right skills and knowledge are in place for both PhT and PT, which will minimize delays and help the department manage the transition more effectively.

### Recommendations

With the PT implementation as an RI in an ambidextrous RT center, loss of critical talent and skill on the PhT side, and PT machine inactivity and its effects on PhT should be carefully considered. To avoid a loss of talent and skill, the organization could recruit and educate EX personnel instead of internal personnel to bypass a loss in knowledge and skill in the PhT team. In addition, it is recommended that all staff be well-trained and confident with all workflows to prevent the rule- and knowledge-based human errors. Furthermore, to prevent organization-based incidents, it is important to ensure that organizational operations (policies, strategies, work procedures, and routines) are well established because these are often underdeveloped when implementing an RI such as PT. In terms of the technology use, it is recommended to be aware that machine inactivity has a larger impact on processes related to PT than on conventional treatment. More specifically, the consequences can differ in magnitude, and interventions should be drawn up for worst-case scenarios.

### Future research

Our study sheds light on the tensions that are mentioned in the literature when addressing ambidexterity, that is, simultaneously conducting business as usual and implementing (radical) innovations. These tensions require attention, but, according to the literature, they do not necessarily result in worse outcomes of the implementation of an innovation.^16^^,^^17^ In our study, we used available data for our analyses; however, for a full evaluation, future research should build on the current list to extend toward qualitative metrics related to the endpoints. Moreover, our research studied only the aftermath of the implementation of PT. A suggestion for future research is to also take preparation steps into account and evaluate steps that can be executed according to implementation research to minimize the negative impact and maximize the benefits of integrating PT in an existing RT center.^17^

## Conclusion

The present study is the first study to give a quantitative representation of the impact of an RI implementation, such as PT, in an ambidextrous context. Although the implementation of PT did not increase the disruption rates for a center that simultaneously conducts PhT, the root causes of the incidents did shift toward more human-related (knowledge-based behavior and rule-based EX) errors in PhT and organizational incidents in PT. No persisting effect on patient satisfaction was identified, but this should be systematically measured through surveys and focus groups in future studies. In terms of technology use, it was found that PT required substantially more quality control, more frequent software and hardware updates, and experienced lower initial uptime compared with PhT. Overall, we can conclude that our lessons learned emphasize the need for adequate preparation before implementation (eg, prevent knowledge loss from existing PhT teams and provide comprehensive training for all workflows), early patient engagement, and careful consideration of the consequences because of machine inactivity (ie, plan for lower initial uptime and coordinate with vendors).

## Appendix Supplementary materials

Appendix E1 Image, application 1

Appendix E2 Image, application 2

Appendix E3 Image, application 3
