# Supplementary material for: Impact of Proton Therapy Implementation on Processes, Patient Satisfaction, and Technology Use in a Radiation Therapy Department
Source: Adv Radiat Oncol. 2025 Dec 25;11(4):101988. doi: 10.1016/j.adro.2025.101988 (PMC12996704; doi:10.1016/j.adro.2025.101988)
Supplement: Appendix E1 [file mmc1.docx]

Appendix E1. Characteristics of radical innovation defined by Poucke^6^, translated to proton therapy (PT). PhT: Photon Therapy; PTC: Proton Therapy Centre.

| Characteristics of radical innovation | Proton therapy |
| --- | --- |
| Unused engineering principles | Completely different and new technology. Protons are accelerated in a cyclotron and react differently in the patient than conventional PhT. |
| Unused scientific principles | The patients’ selection for PT is based on prediction models that estimate the reduction of side effects due to PT compared to PhT, instead of randomized controlled trials. This approach has not been used before. |
| New catchment area | PTCs have to cover a larger catchment area as they were used with their PhT, because only four centers were granted a license to treat all patients in the country. |
| New applications | Extensive scientific research is needed for new indications and applications (e.g., cardiac ablation). |
| Extensive training | New knowledge, new skills, and new competences are required to be able to carry out a successful treatment. |
| Specific management approach | Fundamental change process must be integrated in leadership style due to the ambidextrous ambitions of the organisation. |
