# Supplementary figures and images for: Impact of Proton Therapy Implementation on Processes, Patient Satisfaction, and Technology Use in a Radiation Therapy Department
Source: Adv Radiat Oncol. 2025 Dec 25;11(4):101988. doi: 10.1016/j.adro.2025.101988 (PMC12996704; doi:10.1016/j.adro.2025.101988)

## *Appendix E2. PT and PhT workflow*


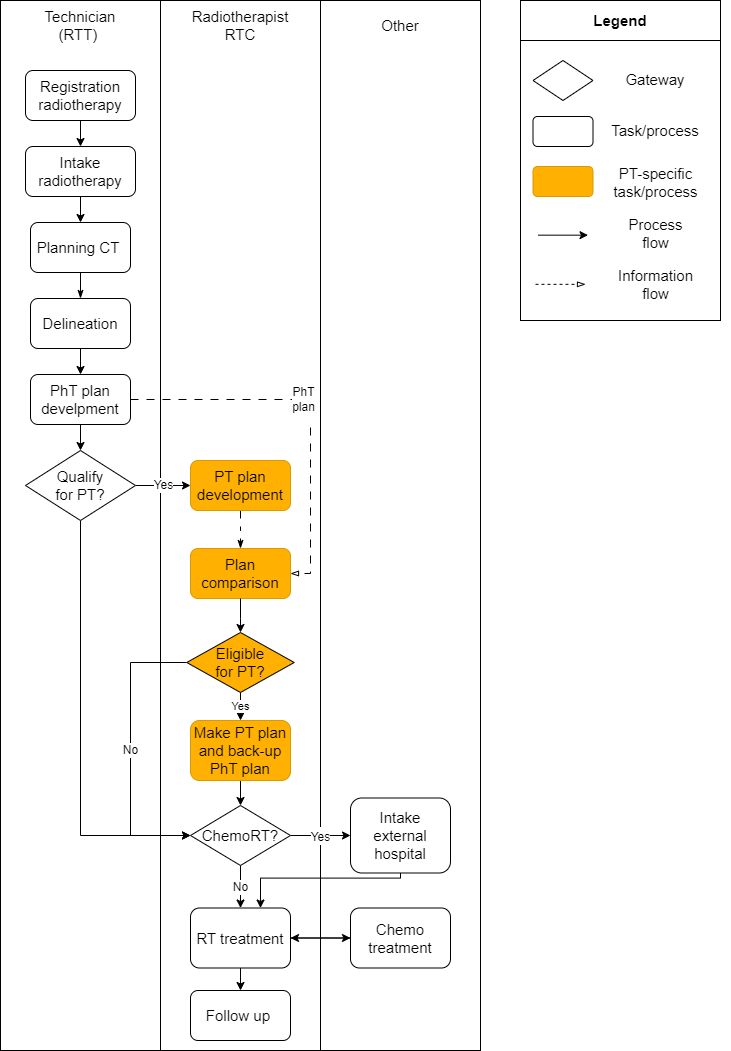

Supplement: Appendix E2 [file mmc2.docx]
