# Supplementary material for: Impact of Proton Therapy Implementation on Processes, Patient Satisfaction, and Technology Use in a Radiation Therapy Department
Source: Adv Radiat Oncol. 2025 Dec 25;11(4):101988. doi: 10.1016/j.adro.2025.101988 (PMC12996704; doi:10.1016/j.adro.2025.101988)
Supplement: Appendix E3 [file mmc3.docx]

## *Appendix E3. Causal impact analyses*

#
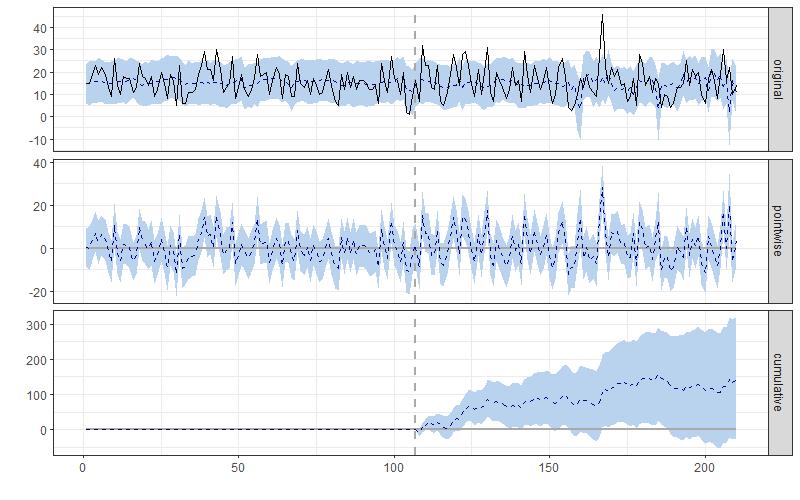


Time in weeks (cumulative)

Process disruptions

Figure C1. Causal impact graph human-related incidents

#
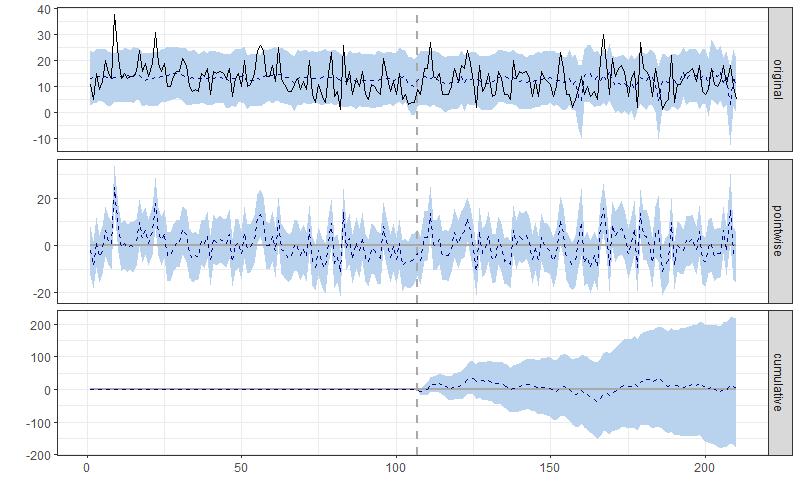


Time in weeks (cumulative)

Process disruptions

Figure C2. Causal impact graph organization-related incidents

#
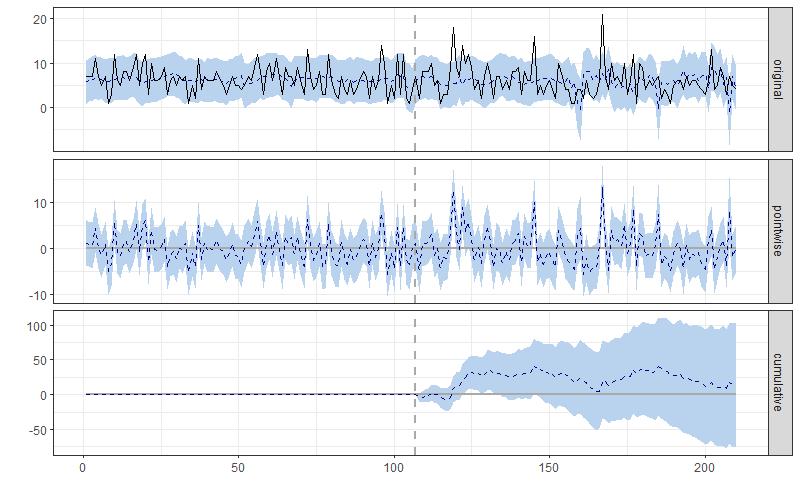


Process disruptions

Time in weeks (cumulative)

Figure C3. Causal impact graph technological related errors
